# 2-Amino­pyrimidinium hydrogen chloranilate monohydrate

**DOI:** 10.1107/S1600536808034740

**Published:** 2008-10-31

**Authors:** Ping Su, Xue-Ying Huang, Xiang-gao Meng

**Affiliations:** a Key Laboratory of Pesticides & Chemical Biology, Ministry of Education, College of Chemistry, Central China Normal University, Wuhan 430079, People’s Republic of China

## Abstract

In the title compound, C_4_H_6_N_3_^+^·C_6_HCl_2_O_4_^−^·H_2_O, anions, cations and water mol­ecules are linked by inter­molecular O—H⋯O, O—H⋯N and N—H⋯O hydrogen bonds into one-dimensional tapes along [111]. These tapes are further linked by weak Cl⋯Cl inter­actions [Cl⋯Cl = 3.394 (2) Å], forming sheets parallel to the (10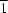) plane.

## Related literature

For background information, see: Aakeröy & Salmon (2005 ▶); Aakeröy *et al.* (2007 ▶); Abrahams *et al.* (2002 ▶); Cueto *et al.* (1992 ▶); Kawata *et al.* (1994 ▶, 1998 ▶). For related crystal structures, see: Meng & Qian (2006 ▶); Min *et al.* (2006 ▶, 2007 ▶); Murata *et al.* (2007 ▶); Wang & Wei (2005 ▶); Yang (2007 ▶); Gaballa *et al.* (2008 ▶); Gotoh *et al.* (2006 ▶, 2007*a* ▶,*b* ▶,*c* ▶); Jia *et al.* (2008 ▶). For bond-length data, see: Allen (2002 ▶); Allen *et al.* (1987 ▶).
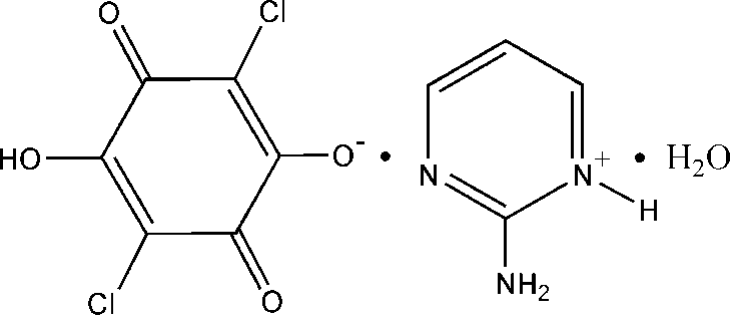


## Experimental

### 

#### Crystal data

C_4_H_6_N_3_^+^·C_6_HCl_2_O_4_^−^·H_2_O
*M* *_r_* = 322.10
Triclinic, 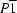

*a* = 6.7969 (5) Å
*b* = 9.4631 (6) Å
*c* = 11.0604 (7) Å
α = 106.074 (1)°
β = 105.892 (1)°
γ = 101.925 (1)°
*V* = 626.01 (7) Å^3^
*Z* = 2
Mo *K*α radiation
μ = 0.54 mm^−1^
*T* = 292 (2) K
0.27 × 0.10 × 0.04 mm

#### Data collection

Bruker SMART APEX CCD area-detector diffractometer
Absorption correction: multi-scan (*SADABS*; Sheldrick, 1996 ▶) *T*_min_ = 0.857, *T*_max_ = 0.979
5691 measured reflections
2121 independent reflections
1348 reflections with *I* > 2σ(*I*)
*R*_int_ = 0.071

#### Refinement

*R*[*F* ^2^ > 2σ(*F* ^2^)] = 0.078
*wR*(*F* ^2^) = 0.197
*S* = 0.97
2121 reflections
199 parameters
6 restraints
H atoms treated by a mixture of independent and constrained refinement
Δρ_max_ = 0.66 e Å^−3^
Δρ_min_ = −0.45 e Å^−3^

### 

Data collection: *SMART* (Bruker, 2007 ▶); cell refinement: *SAINT-Plus* (Bruker, 2007 ▶); data reduction: *SAINT-Plus*; program(s) used to solve structure: *SHELXS97* (Sheldrick, 2008 ▶); program(s) used to refine structure: *SHELXL97* (Sheldrick, 2008 ▶); molecular graphics: *PLATON* (Spek, 2003 ▶); software used to prepare material for publication: *PLATON*.

## Supplementary Material

Crystal structure: contains datablocks global, I. DOI: 10.1107/S1600536808034740/lh2716sup1.cif

Structure factors: contains datablocks I. DOI: 10.1107/S1600536808034740/lh2716Isup2.hkl

Additional supplementary materials:  crystallographic information; 3D view; checkCIF report

## Figures and Tables

**Table 1:** Hydrogen-bond geometry (Å, °)

*D*—H⋯*A*	*D*—H	H⋯*A*	*D*⋯*A*	*D*—H⋯*A*
O4—H4⋯O3	0.83 (2)	2.16 (6)	2.651 (5)	118 (5)
O4—H4⋯N1^i^	0.83 (2)	2.07 (4)	2.795 (6)	146 (6)
O5—H5*A*⋯O2	0.82 (4)	2.09 (3)	2.872 (5)	156 (6)
O5—H5*A*⋯O1	0.82 (4)	2.34 (4)	2.859 (5)	121 (4)
O5—H5*B*⋯O2^ii^	0.82 (4)	2.09 (4)	2.830 (5)	150 (5)
N3—H3*A*⋯O5	0.86 (2)	2.02 (3)	2.815 (6)	153 (5)
N2—H2⋯O1	0.84 (5)	1.98 (5)	2.793 (6)	163 (5)
N3—H3*B*⋯O3^iii^	0.86 (2)	2.17 (4)	2.953 (6)	151 (5)

Symmetry codes: (i) 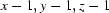; (ii) 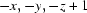; (iii) 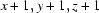.

**Fig. 1.**
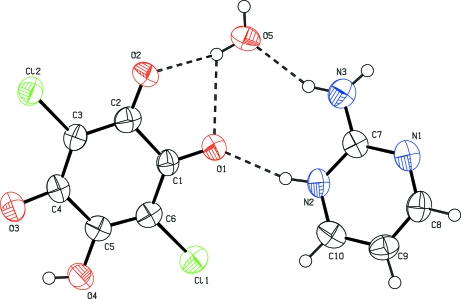
Molecular structure of (I), showing the atom-numbering scheme. Displacement ellipsoids are drawn at the 30% probability level. H-bonds are shown in dashed lines.

**Fig. 2.**
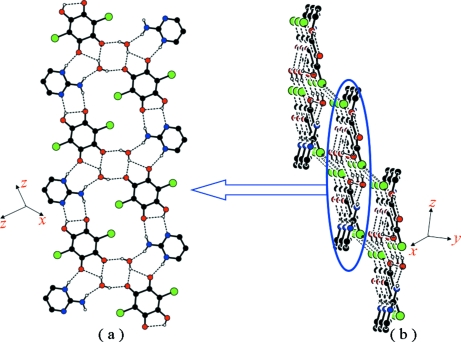
Part of the crystal structure of (I), showing the formation of the one-dimensional tape (a) linked by intermolecular O-H···O and N-H···O hydrogen bonds parallel to the [111] direction and the two-dimensional sheet (b) linked by Cl···Cl interactin. For the sake of clarity, H atoms not involved in the motif have been omitted from the drawing.

## supplementary crystallographic information

### Comment

Chloranilic acid (CA) can be regarded as a strong organic acid (p*K*_a1_= 1.38; p*K*_a2_ = 2.98) which can release its two hydroxyl protons easily. For this reason, CA is often used as a bridge ligand in the synthesis of metal coordination complexes (Kawata *et al.*, 1994; Kawata *et al.*, 1998; Abrahams *et al.*, 2002; Cueto *et al.*, 1992; Min *et al.*, 2006; Min *et al.*, 2007) or used as a cocrystal agent in the construction of supramolecular structure based on hydrogen-bonds (Gotoh *et al.*, 2006, 2007*a*, 2007*b* and 2007*c*; Murata *et al.*, 2007; Gaballa *et al.*, 2008; Jia *et al.*, 2008). As part of our continuing studies on the synthesis of co-crystal or organic salts involved CA (Meng & Qian, 2006), we report here the crystal structure of the title compound (I) which was obtained by mixing equivalent amount of CA and 2-aminopyrimidine (2-APy) in 95% methanol solution at room temperature.

In (I), one of the CA hydroxyl protons is transferred to a pyrimidine N atom, forming a 1:1 organic adduct with one water molecule being incorporated into the crystal lattice (Fig. 1). According to the definitions of co-crystal and organic salt proposed by Aakeröy and Salmon (2005), complex (I) can be considered as an organic salt. The bond lengths and bonds angles in the CA^-^ anion are comparable with those from some analogues (Wang & Wei, 2005; Yang, 2007). In the 2-APy^+^ cation, the angles of C7—N1—C8 and C7—N2—C10 [116.5 (1)° and 122.2 (1)°, respectively] are both consistent with the magnitude of C—N—C angles in unprotonated and protonated pyridine molecules [116.3 (16)° and 122.4 (16)°, respectively] (Allen *et al.*, 1987; Allen, 2002). All other geomtric parameters in the structure are as expected.

In the crystal structure, intermolecular O–H···O and  N–H···O hydrogen bonds (Table 1), link the components of (I) into one-dimensional tapes along [111] (Fig.2). In addition, neighbouring tapes are linked by weak Cl···Cl interactions [Cl···Cl^i^ = 3.394 (2) Å, see: Aakeröy *et al.*, 2007); symmetry code: (i) x, y+1, z)] resulting in two-dimensional sheets parallel to the (10-1) plane.

### Experimental

All the reagents and solvents were used as obtained without further purification. Equivalent molar amount of chloranilic acid (1 mmol, 210 mg) and 2-aminopyimidine (1 mmol, 9.5 mg) were dissolved in 95% methanol (20 ml). The mixture was stirred for half an hour at ambient temperature and then filtered. The resulting red solution was kept in air for two week. Plate-like crystals of (I) suitable for single-crystal X-ray diffraction analysis were grown at the bottom of the vessel by slow evaporation of the solution.

### Refinement

H atoms bonded to C atoms were located in difference maps and subsequently treated as riding modes, with C–H=0.93 Å and *U*_iso_(H) = 1.2*U*_eq_(C). H atoms bonded to N and O atoms were also found in difference maps, with the constraints of N—H =0.86 (2)Å and O—H =0.82 (2) Å, and the *U*_iso_(H) values being set k times of their carrier atoms (k=1.2 for N and 1.5 for O atoms)

### Crystal data

**Table:**

C_4_H_6_N_3_^+^·C_6_HCl_2_O_4_^−^·H_2_O	*Z* = 2
*M**_r_* = 322.10	*F*(000) = 328
Triclinic, *P*1	*D*_x_ = 1.709 Mg m^−^^3^
Hall symbol: -P 1	Mo *K*α radiation, λ = 0.71073 Å
*a* = 6.7969 (5) Å	Cell parameters from 1004 reflections
*b* = 9.4631 (6) Å	θ = 2.2–25.2°
*c* = 11.0604 (7) Å	µ = 0.54 mm^−^^1^
α = 106.074 (1)°	*T* = 292 K
β = 105.892 (1)°	Plate, red
γ = 101.925 (1)°	0.27 × 0.10 × 0.04 mm
*V* = 626.01 (7) Å^3^	

### Data collection

**Table:**

Bruker SMART APEX CCD area-detector diffractometer	2121 independent reflections
Radiation source: fine focus sealed Siemens Mo tube	1348 reflections with *I* > 2σ(*I*)
graphite	*R*_int_ = 0.071
0.3° wide ω exposures scans	θ_max_ = 25.0°, θ_min_ = 2.4°
Absorption correction: multi-scan (SADABS; Sheldrick, 1996)	*h* = −7→8
*T*_min_ = 0.857, *T*_max_ = 0.979	*k* = −11→11
5691 measured reflections	*l* = −13→13

### Refinement

**Table:**

Refinement on *F*^2^	Primary atom site location: structure-invariant direct methods
Least-squares matrix: full	Secondary atom site location: difference Fourier map
*R*[*F*^2^ > 2σ(*F*^2^)] = 0.078	Hydrogen site location: inferred from neighbouring sites
*wR*(*F*^2^) = 0.197	H atoms treated by a mixture of independent and constrained refinement
*S* = 0.97	*w* = 1/[σ^2^(*F*_o_^2^) + (0.1114*P*)^2^] where *P* = (*F*_o_^2^ + 2*F*_c_^2^)/3
2121 reflections	(Δ/σ)_max_ < 0.001
199 parameters	Δρ_max_ = 0.66 e Å^−^^3^
6 restraints	Δρ_min_ = −0.45 e Å^−^^3^

### Special details

**Table:**

Geometry. All esds (except the esd in the dihedral angle between two l.s. planes) are estimated using the full covariance matrix. The cell esds are taken into account individually in the estimation of esds in distances, angles and torsion angles; correlations between esds in cell parameters are only used when they are defined by crystal symmetry. An approximate (isotropic) treatment of cell esds is used for estimating esds involving l.s. planes.
Refinement. Refinement of F^2^ against ALL reflections. The weighted R-factor wR and goodness of fit S are based on F^2^, conventional R-factors R are based on F, with F set to zero for negative F^2^. The threshold expression of F^2^ > 2sigma(F^2^) is used only for calculating R-factors(gt) etc. and is not relevant to the choice of reflections for refinement. R-factors based on F^2^ are statistically about twice as large as those based on F, and R- factors based on ALL data will be even larger.

### Fractional atomic coordinates and isotropic or equivalent isotropic displacement parameters (Å^2^)

**Table:**

	*x*	*y*	*z*	*U*_iso_*/*U*_eq_	
C1	−0.1247 (7)	0.1172 (6)	0.1769 (5)	0.0361 (12)	
C2	−0.1473 (7)	−0.0408 (6)	0.2001 (5)	0.0425 (13)	
C3	−0.2335 (8)	−0.1755 (5)	0.0838 (5)	0.0378 (12)	
C4	−0.3014 (7)	−0.1738 (6)	−0.0449 (5)	0.0358 (12)	
C5	−0.2867 (7)	−0.0171 (6)	−0.0623 (5)	0.0372 (12)	
C6	−0.2029 (7)	0.1163 (6)	0.0438 (5)	0.0363 (12)	
Cl1	−0.1870 (2)	0.29304 (15)	0.02393 (13)	0.0479 (5)	
Cl2	−0.2558 (2)	−0.35198 (15)	0.10632 (14)	0.0500 (5)	
O1	−0.0385 (6)	0.2339 (4)	0.2793 (3)	0.0506 (10)	
O2	−0.0871 (6)	−0.0336 (4)	0.3183 (3)	0.0547 (11)	
O3	−0.3780 (6)	−0.2884 (4)	−0.1520 (4)	0.0513 (10)	
O4	−0.3561 (6)	−0.0196 (4)	−0.1857 (4)	0.0509 (10)	
H4	−0.399 (10)	−0.110 (3)	−0.238 (5)	0.076*	
C7	0.3286 (7)	0.6009 (6)	0.5065 (5)	0.0365 (12)	
C8	0.5278 (8)	0.8089 (6)	0.4821 (6)	0.0462 (14)	
H8	0.6301	0.9059	0.5206	0.055*	
C9	0.4403 (9)	0.7485 (6)	0.3461 (6)	0.0481 (14)	
H9	0.4801	0.8020	0.2933	0.058*	
C10	0.2900 (8)	0.6044 (7)	0.2895 (5)	0.0468 (14)	
H10	0.2244	0.5573	0.1967	0.056*	
N1	0.4796 (6)	0.7413 (5)	0.5644 (4)	0.0447 (11)	
N2	0.2407 (6)	0.5338 (5)	0.3717 (4)	0.0378 (11)	
H2	0.162 (9)	0.442 (6)	0.329 (5)	0.045*	
N3	0.2768 (7)	0.5294 (6)	0.5858 (5)	0.0504 (12)	
H3A	0.200 (8)	0.434 (3)	0.548 (5)	0.060*	
H3B	0.337 (9)	0.579 (6)	0.671 (2)	0.060*	
O5	0.0043 (6)	0.2393 (4)	0.5451 (3)	0.0493 (10)	
H5A	−0.050 (10)	0.171 (5)	0.468 (2)	0.074*	
H5B	0.021 (10)	0.205 (6)	0.606 (3)	0.074*	

### Atomic displacement parameters (Å^2^)

**Table:**

	*U*^11^	*U*^22^	*U*^33^	*U*^12^	*U*^13^	*U*^23^
C1	0.026 (3)	0.037 (3)	0.041 (3)	0.006 (2)	0.012 (2)	0.009 (3)
C2	0.024 (3)	0.053 (4)	0.050 (3)	0.008 (2)	0.011 (2)	0.023 (3)
C3	0.034 (3)	0.034 (3)	0.046 (3)	0.013 (2)	0.015 (2)	0.013 (3)
C4	0.020 (3)	0.042 (3)	0.040 (3)	0.011 (2)	0.010 (2)	0.006 (3)
C5	0.027 (3)	0.047 (3)	0.042 (3)	0.016 (2)	0.016 (2)	0.016 (3)
C6	0.023 (3)	0.045 (3)	0.048 (3)	0.015 (2)	0.015 (2)	0.020 (3)
Cl1	0.0427 (8)	0.0484 (9)	0.0582 (9)	0.0174 (6)	0.0181 (7)	0.0246 (7)
Cl2	0.0507 (9)	0.0413 (8)	0.0540 (9)	0.0113 (6)	0.0153 (7)	0.0161 (7)
O1	0.058 (2)	0.044 (2)	0.040 (2)	0.0055 (19)	0.0164 (19)	0.0100 (19)
O2	0.070 (3)	0.049 (2)	0.034 (2)	0.010 (2)	0.0066 (19)	0.0145 (18)
O3	0.052 (2)	0.046 (2)	0.044 (2)	0.0126 (19)	0.0096 (19)	0.0068 (19)
O4	0.062 (3)	0.046 (2)	0.038 (2)	0.016 (2)	0.0110 (19)	0.0117 (17)
C7	0.024 (3)	0.045 (3)	0.043 (3)	0.018 (2)	0.013 (2)	0.012 (3)
C8	0.026 (3)	0.047 (3)	0.059 (4)	0.006 (2)	0.015 (3)	0.013 (3)
C9	0.044 (3)	0.057 (4)	0.053 (4)	0.019 (3)	0.019 (3)	0.029 (3)
C10	0.040 (3)	0.062 (4)	0.043 (3)	0.021 (3)	0.017 (3)	0.017 (3)
N1	0.031 (2)	0.048 (3)	0.051 (3)	0.016 (2)	0.010 (2)	0.011 (2)
N2	0.024 (2)	0.037 (2)	0.044 (3)	0.0089 (18)	0.0065 (19)	0.007 (2)
N3	0.041 (3)	0.052 (3)	0.058 (3)	0.014 (2)	0.017 (3)	0.020 (3)
O5	0.051 (2)	0.059 (2)	0.040 (2)	0.021 (2)	0.013 (2)	0.0214 (19)

### Geometric parameters (Å, °)

**Table:**

C1—O1	1.234 (6)	C7—N2	1.347 (6)
C1—C6	1.419 (7)	C7—N1	1.354 (6)
C1—C2	1.569 (7)	C8—N1	1.321 (6)
C2—O2	1.236 (5)	C8—C9	1.355 (7)
C2—C3	1.412 (7)	C8—H8	0.9300
C3—C4	1.377 (7)	C9—C10	1.377 (7)
C3—Cl2	1.738 (5)	C9—H9	0.9300
C4—O3	1.252 (6)	C10—N2	1.341 (6)
C4—C5	1.533 (7)	C10—H10	0.9300
C5—O4	1.308 (6)	N2—H2	0.84 (5)
C5—C6	1.346 (7)	N3—H3A	0.86 (2)
C6—Cl1	1.732 (5)	N3—H3B	0.86 (2)
O4—H4	0.83 (2)	O5—H5A	0.82 (4)
C7—N3	1.323 (6)	O5—H5B	0.82 (4)
			
O1—C1—C6	125.3 (5)	N3—C7—N1	118.2 (5)
O1—C1—C2	115.6 (4)	N2—C7—N1	120.6 (4)
C6—C1—C2	119.1 (5)	N1—C8—C9	125.3 (5)
O2—C2—C3	127.1 (5)	N1—C8—H8	117.3
O2—C2—C1	116.4 (5)	C9—C8—H8	117.3
C3—C2—C1	116.5 (4)	C8—C9—C10	117.1 (5)
C4—C3—C2	123.5 (4)	C8—C9—H9	121.4
C4—C3—Cl2	118.9 (4)	C10—C9—H9	121.4
C2—C3—Cl2	117.6 (4)	N2—C10—C9	118.2 (5)
O3—C4—C3	126.8 (5)	N2—C10—H10	120.9
O3—C4—C5	115.2 (4)	C9—C10—H10	120.9
C3—C4—C5	118.1 (5)	C8—N1—C7	116.5 (5)
O4—C5—C6	121.7 (5)	C10—N2—C7	122.2 (5)
O4—C5—C4	116.6 (4)	C10—N2—H2	112 (3)
C6—C5—C4	121.8 (4)	C7—N2—H2	126 (3)
C5—C6—C1	121.0 (5)	C7—N3—H3A	117 (4)
C5—C6—Cl1	121.7 (4)	C7—N3—H3B	117 (4)
C1—C6—Cl1	117.4 (4)	H3A—N3—H3B	126 (5)
C1—O1—H2	135.7 (14)	H3A—O5—H5A	112 (4)
C2—O2—H5A	118.4 (13)	H3A—O5—H5B	126 (4)
C5—O4—H4	109 (4)	H5A—O5—H5B	114 (3)
N3—C7—N2	121.2 (5)		
			
O1—C1—C2—O2	3.6 (7)	O4—C5—C6—Cl1	1.4 (7)
C6—C1—C2—O2	−176.2 (4)	C4—C5—C6—Cl1	−179.9 (3)
O1—C1—C2—C3	−176.3 (4)	O1—C1—C6—C5	177.2 (5)
C6—C1—C2—C3	3.9 (7)	C2—C1—C6—C5	−3.0 (7)
O2—C2—C3—C4	178.5 (5)	O1—C1—C6—Cl1	−3.0 (7)
C1—C2—C3—C4	−1.7 (7)	C2—C1—C6—Cl1	176.8 (3)
O2—C2—C3—Cl2	−1.0 (7)	C6—C1—O1—H2	−38 (2)
C1—C2—C3—Cl2	178.8 (3)	C2—C1—O1—H2	143 (2)
C2—C3—C4—O3	179.6 (5)	C3—C2—O2—H5A	−163 (2)
Cl2—C3—C4—O3	−0.9 (7)	C1—C2—O2—H5A	17 (2)
C2—C3—C4—C5	−1.4 (7)	N1—C8—C9—C10	−0.4 (8)
Cl2—C3—C4—C5	178.1 (3)	C8—C9—C10—N2	−0.1 (8)
O3—C4—C5—O4	0.4 (6)	C9—C8—N1—C7	−0.7 (8)
C3—C4—C5—O4	−178.8 (4)	N3—C7—N1—C8	179.8 (5)
O3—C4—C5—C6	−178.4 (4)	N2—C7—N1—C8	2.4 (7)
C3—C4—C5—C6	2.5 (7)	C9—C10—N2—C7	1.9 (8)
O4—C5—C6—C1	−178.8 (4)	N3—C7—N2—C10	179.6 (5)
C4—C5—C6—C1	−0.1 (7)	N1—C7—N2—C10	−3.1 (7)

### Hydrogen-bond geometry (Å, °)

**Table:**

*D*—H···*A*	*D*—H	H···*A*	*D*···*A*	*D*—H···*A*
O4—H4···O3	0.83 (2)	2.16 (6)	2.651 (5)	118 (5)
O4—H4···N1^i^	0.83 (2)	2.07 (4)	2.795 (6)	146 (6)
O5—H5A···O2	0.82 (4)	2.09 (3)	2.872 (5)	156 (6)
O5—H5A···O1	0.82 (4)	2.34 (4)	2.859 (5)	121 (4)
O5—H5B···O2^ii^	0.82 (4)	2.09 (4)	2.830 (5)	150 (5)
N3—H3A···O5	0.86 (2)	2.02 (3)	2.815 (6)	153 (5)
N2—H2···O1	0.84 (5)	1.98 (5)	2.793 (6)	163 (5)
N3—H3B···O3^iii^	0.86 (2)	2.17 (4)	2.953 (6)	151 (5)

Symmetry codes: (i) *x*−1, *y*−1, *z*−1; (ii) −*x*, −*y*, −*z*+1; (iii) *x*+1, *y*+1, *z*+1.

## References

[bb1] Aakeröy, C. B., Fasulo, M., Schultheiss, N., Desper, J. & Moore, C. (2007). *J. Am. Chem. Soc.* **129**, 13772–3773. 10.1021/ja073201c 17956090

[bb2] Aakeröy, C. B. & Salmon, D. J. (2005). *CrystEngComm*, **7**, 439–448. 10.1039/b811322j PMC2748950 20046916

[bb3] Abrahams, B. F., Coleiro, J., Ha, K., Hoskins, B. F., Drchard, S. D. & Robson, R. (2002). *J. Chem. Soc. Dalton Trans.* pp. 1586–1594.

[bb4] Allen, F. H. (2002). *Acta Cryst.* B**58**, 380–388. 10.1107/s0108768102003890 12037359

[bb5] Allen, F. H., Kennard, Q., Waterson, D. G., Brammer, L., Orpen, A. G. & Taylor, R. (1987). *J. Chem. Soc. Perkin Trans. 2*, pp. S1–19.

[bb6] Bruker (2007). *SAINT-Plus* and *SMART* Bruker AXS Inc., Madison, Wisconsin, USA.

[bb7] Cueto, S., Straumann, H.-P., Rys, P., Petter, W., Gramlich, V. & Rys, F. S. (1992). *Acta Cryst.* C**48**, 458–460.

[bb8] Gaballa, A. S., Wagner, C., Teleb, S. M., Nour, E. M., Elmosallamy, M. A. F., Kaluderovic, G. N., Schmidt, H. & Steinborn, D. (2008). *J. Mol. Struct.* **876**, 301–307.

[bb9] Gotoh, K., Ishikawa, R. & Ishida, H. (2007*a*). *Acta Cryst.* E**63**, o4433.

[bb10] Gotoh, K., Ishikawa, R. & Ishida, H. (2007*c*). *Acta Cryst.* E**63**, o4518.

[bb11] Gotoh, K., Nagoshi, H. & Ishida, H. (2007*b*). *Acta Cryst.* E**63**, o4295.

[bb12] Gotoh, K., Ishikawa, R. & Ishida, H. (2006). *Acta Cryst.* E**62**, o4738–o4740.

[bb13] Jia, L.-H., Mu, Z.-E. & Liu, Z.-L. (2008). *Acta Cryst.* E**64**, o32. 10.1107/S1600536807048374 PMC2914992 21200881

[bb14] Kawata, S., Kitagawa, S., Kondo, M., Furuchi, I. & Munakata, M. (1994). *Angew. Chem. Int. Ed.* **33**, 1759–1761.

[bb15] Kawata, S., Kitagawa, S., Kumagai, H., Ishiyama, T., Honda, K., Tobita, H., Adachi, K. & Katada, M. (1998). *Chem. Mater.* **10**, 3902–3912.

[bb16] Meng, X.-G. & Qian, J.-L. (2006). *Acta Cryst.* E**62**, o4178–o4180.

[bb17] Min, K. S., DiPasquale, A. G., Golen, J. A., Rheingold, A. L. & Miller, J. S. (2007). *J. Am. Chem. Soc.* **129**, 2360–2368. 10.1021/ja067208q 17269771

[bb18] Min, K. S., Rheingold, A. L., DiPasquale, A. & Miller, J. S. (2006). *Inorg. Chem.* **45**, 6135–6137. 10.1021/ic061076k 16878921

[bb19] Murata, T., Morita, Y., Yakiyama, Y., Fukui, K., Yamochi, H., Saito, G. & Nakasuji, K. (2007). *J. Am. Chem. Soc.* **129**, 10837–10846. 10.1021/ja072607m 17696346

[bb20] Sheldrick, G. M. (1996). *SADABS* Bruker AXS Inc., Madison, Wisconsin, USA.

[bb21] Sheldrick, G. M. (2008). *Acta Cryst.* A**64**, 112–122. 10.1107/S0108767307043930 18156677

[bb22] Spek, A. L. (2003). *J. Appl. Cryst.* **36**, 7–13.

[bb23] Wang, Z.-L. & Wei, L.-H. (2005). *Acta Cryst.* E**61**, o3129–o3130.

[bb24] Yang, D.-J. (2007). *Acta Cryst.* E**63**, o2600.

